# Characterization of Agarolytic Pathway in a Terrestrial Bacterium *Cohnella* sp. LGH

**DOI:** 10.3389/fmicb.2022.828687

**Published:** 2022-03-31

**Authors:** Gen Li, Rui Guo, Shuqi Wu, Si Cheng, Jiaqi Li, Zhenzhen Liu, Wangliang Xie, Xiaolin Sun, Qiuyi Zhang, Zihan Li, JiaZheng Xu, Jun Wu, Zhong Wei, Feng Hu

**Affiliations:** ^1^Soil Ecology Lab, College of Resources and Environmental Sciences, Nanjing Agricultural University, Nanjing, China; ^2^Jiangsu Provincial Key Lab for Organic Solid Waste Utilization, Key Lab of Plant Immunity, Nanjing, China; ^3^College of Resources and Environmental Sciences, Nanjing Agricultural University, Nanjing, China

**Keywords:** agarose, agarotetraose, neoagarotriose, endo-type β-agarase, α-NAOS hydrolase, agarolytic β-galactosidase, enzymatic properties, terrestrial bacterium *Cohnella* sp. LGH

## Abstract

Previously, we have reported that an endo-type β-agarase AgaW was responsible for the hydrolysis of agarose into the major product neoagarotetraose in a terrestrial agar-degrading bacterium *Cohnella* sp. LGH. Here, we identify and characterize the following depolymerization pathway in strain LGH through the genomic and enzymatic analysis. In the pathway, neoagarotetraose was depolymerized by a novel α-neoagarooligosaccharide (NAOS) hydrolase CL5012 into 3,6-anhydro-α-L-galactose (L-AHG) and agarotriose; Agarotriose was further depolymerized by a novel agarolytic β-galactosidase CL4994 into D-galactose and neoagarobiose; Neoagarobiose was finally depolymerized by CL5012 into L-AHG and D-galactose. Although α-agarase has not been identified in strain LGH, the combined action of CL5012 and CL4994 unexpectedly plays a critical role in the depolymerization of agarotetraose, one theoretical product of α-agarase hydrolysis of agarose. In this pathway, agarotetraose was depolymerized by CL4994 into D-galactose and neoagarotriose; Neoagarotriose was then depolymerized by CL5012 into L-AHG and agarobiose. Furthermore, another novel endo-type β-agarase CL5055 was identified as an isozyme of AgaW with different pH preference in the hydrolysis of agarose into α-NAOSs. Strain LGH seemed to lack a common exo-type β-agarase responsible for the direct depolymerization of agarose or neoagarooligosaccharide into neoagarobiose. These results highlight the diversity of agarolytic manner in bacteria and provide a novel insight on the diversity of agarolytic pathways.

## Introduction

Agarose is the main carbohydrate component of the cell wall in red algae. It is a linear polysaccharide and consists of two alternating monosaccharides, 3-*O*-linked β-D-galactose and 4-*O*-linked 3,6-anhydro-α-L-galactose (L-AHG) (Chi et al., 2012; Jiang et al., 2020a). D-galactose is a common monosaccharide that can be utilized by most microorganisms through the Leloir pathway and DeLey-Doudoroff (DD) pathway, while L-AHG cannot be catabolized by conventional microorganisms, such as *Saccharomyces cerevisiae* and *Escherichia coli* (Wong and Yao, 1994; Holden et al., 2003; Kim et al., 2012; Yun et al., 2016). Agarose oligosaccharides are divided into neoagarooligosaccharides (NAOSs) and agarooligosaccharides (AOSs) according to their non- reducing ends on L-AHG and D-galactose, respectively (Jiang et al., 2020a). Recently, red algae have attracted considerable attention and have been deemed as an ideal primary material of biomass for its renewable property and large yields (John et al., 2011; Kim et al., 2012). Furthermore, agarose, the typical component of red algae, is considered a significant carbon source for heterotrophic microorganisms for their lack of recalcitrant substrates (Arnosti et al., 2014). Thus, the catabolic process of polysaccharides is an important part of global carbon cycle (Reintjes et al., 2017). Due to the biological activities of agaro-oligosaccharides, such as anti-inflammatory (Kim et al., 2017; Ma et al., 2019), anti-diabetic (Park et al., 2020), and anti-cariogenic (Yun et al., 2017), agarose is utilized as a raw material in the production of biochemicals (Veerakumar and Manian, 2018; Park et al., 2020). Therefore, analysis of the agarose catabolism process by microorganisms not only has significance on f how microorganisms participate in the carbon cycle by utilizing agarose, but also offers a molecular basis for the industrial production of oligomeric sugars and biofuels.

To date, several agar-degrading bacteria have been reported (Jiang et al., 2020a; Yu et al., 2020; Pathiraja et al., 2021). The complete agarolytic pathways responsible for the depolymerization of agarose into monosaccharides have been characterized in a few bacteria, such as *Vibrio* sp. EJY3, *Agarivorans gilvus* WH0801, *Colwellia echini* A3^T^, and *Paraglaciecola hydrolytica* S66^T^ from marine environments (Schultz-Johansen et al., 2018; Yang et al., 2018; Yu et al., 2020; Pathiraja et al., 2021), *Streptomyces coelicolor* A3(2) from terrestrial environments (Jiang et al., 2020b), and *Bacteroides uniformis* NP1 from human intestines (Pluvinage et al., 2018). The common agarose depolymerization pathway was composed of an endo-type β-agarase, an exo-type β-agarase and an α-NAOS hydrolase (Yu et al., 2020; Pathiraja et al., 2021). The endo-type β-agarase usually performed the first step to depolymerize agarose into α-NAOSs, such as AgWH50A from *A. gilvus* WH0801 (Liu et al., 2014a), AgaA from *Zobellia galactanivorans* (Jam et al., 2005), and Aga2 from *Cellulophaga omnivescoria* W5C (Ramos et al., 2018). The exo-type β-agarase then depolymerized the short-chain α-NAOSs as neoagarotetraose, neoagarohexaose, or the long-chain α-NAOSs into neoagarobiose, such as AgWH50C from *A*. *gilvus* WH0801 (Liu et al., 2014b), Aga50D from *Saccharophagus degradans* 2-40^T^ (Kim et al., 2010), and DagB from *S*. *coelicolor* A3(2) (Temuujin et al., 2011). The α-NAOS hydrolase further recognized and cleaved α-1,3 linkage of neoagarobiose into monosaccharides, such as Ahg558 from *Gayadomonas joobiniege* G7 (Asghar et al., 2019), AgaWH117 from *A. gilvus* WH0801 (Liu et al., 2016), and BpGH117 from *Bacteroides plebeius* (Hehemann et al., 2012). Recently, the first auxiliary agarolytic pathway (Aux I) has been reported from *Vibrio* sp. EJY3, in which the α-NAOS hydrolase VejGH117 depolymerized α-NAOS into L-AHG and AOSs and then the agarolytic β-galactosidase (ABG) VejABG cleaved β-1,4 linkage of AOSs into D-galactose and shorter-chain α-NAOSs (Yu et al., 2020). At present, several ABGs have been reported from bacteria, such as VejABG from *Vibrio* sp. EJY3 (Lee et al., 2014), AgWH2A from *A. gilvus* WH0801 (Yang et al., 2018), and GH2C from *B. uniformis* NP1 (Pluvinage et al., 2018). In 2021, the second auxiliary agarolytic pathway (Aux II) was identified and characterized from *C*. *echini* A3^T^, in which the α-agarase Ce2835 depolymerized the short-chain α-NAOS neoagarohexaose into neoagarotriose and shorter-chain AOS agarotriose (Pathiraja et al., 2021). The exo-type β-agarase Ce2842 further depolymerized neoagarotriose into L-AHG and neoagarobiose, while the ABG Ce2828 further depolymerized agarotriose into D-galactose and neoagarobiose. Usually, the common agarolytic pathway was present in nearly all agar-degrading bacteria, while the Aux I or the Aux II agarolytic pathway may be absent in some of them (Pathiraja et al., 2021). Besides these agarolytic pathways, agarose could also be depolymerized directly by α-agarase, such as Ce2835 and AgaWS5, into agarooligosaccharides, such as agarotetraose and agarohexaose (Lee et al., 2019; Pathiraja et al., 2021), and Pathiraja also characterized the further depolymerization pathway (Pathiraja et al., 2021). Only an agarolytic β-galactosidase VejABG being responsible for even number AOSs degradation was reported (Lee et al., 2014).

An agar-degrading bacterium *Cohnella* sp. LGH was previously isolated from soil and an endo-type β-agarase AgaW responsible for the hydrolysis of agarose into the major product neoagarotetraose was characterized (Li et al., 2015). Here, we sequenced and analyzed the genome of strain LGH for investigation of its following agarolytic pathway. The functions of the candidate proteins were determined and the biochemical properties of the enzymes involved in the following agarolytic pathway were also characterized. We found that the combination of a novel α-NAOS hydrolase CL5012 and a novel ABG CL4994 could depolymerize not only neoagarotetraose into monosaccharides, but also agarotetraose, one theoretical product of α-agarase hydrolysis of agarose, into monosaccharides and agarobiose. Based on these findings, we proposed a model of agarose depolymerization pathways in strain LGH. Our results indicate the diversity in bacterial agarose catabolism and offer new insight into agarose utilization in terrestrial bacteria.

## Materials and Methods

### Biochemical Reagents

Agarose and a series of agaro-oligosaccharides were purchased from Qingdio BZ Oligo Biotech Co., Ltd., China. Other chemical reagents used in this study were all of analytical grade and obtained from Shanghai Sangon Biological Engineering Technology and Service Co., Ltd., China. All enzymes, competent cells, and kits necessary for DNA and RNA manipulation were purchased from Takara Biotechnology (Dalian) Co., Ltd., China. The Amicon ultra-2 and ultra-15 centrifugal filter unit (3-kDa cutoff size) was purchased from Millipore. Plasmid pET29a (+) and pBAD-HisA were stored in Soil Ecology Lab. *A. gilvus* WH0801 was purchased from CGMCC and gene sequences of AgaWS5, VejAHGD, and VejACI were synthesized by Sangon Biological Engineering Technology and Service Co., Ltd.

### Strain and Medium

*Cohnella* sp. LGH was isolated from farmland soil in NanJing, China. LB medium contained 1% NaCl, 1% Tryptone, and 0.5% Yeast extract. MM medium contained 0.1% NaCl, 0.2% (NH_4_)_2_SO_4_, 0.15% K_2_HPO_4_, 0.05% KH_2_PO_4_, 0.01% CaCl_2_, 0.01% MgSO_4_, and pH 7.2. MS medium contained MM medium and 0.5% glucose. NAOS medium contained MM medium and 0.5% hydrolysis product of AgaW acting on agarose.

### Conversion of Neoagarooligosaccharides by Strain LGH and Its Crude Enzyme

The NAOSs were prepared by β-agarase AgaW as previously described (Li et al., 2015). Strain LGH grew in LB medium in a shark (250 rpm) at 30°C for 24 h, and then inoculated (1%) to NAOS medium. The cell density was measured spectrophotometrically at 600 nm with regular intervals. Meanwhile, the residual NAOSs of liquid medium were determined by TLC at intervals of 24 h. To prepare crude enzyme extracts, the LGH cells were centrifuged and collected after culturing with NAOS medium for 96 h. The cell pellet was washed three times by 10 mM potassium phosphate buffer (pH 7.5). The resuspended cells were lysed by sonication, and lyzed cells were centrifuged at 15,000 × *g* for 30 min. The supernatant was desalted by a CommaX™ G-25 column (Biocomma Co., Ltd., China) to obtain crude enzyme. The crude enzyme (10 μl) was incubated with 10 μl of 2 mg ml^–1^ NAOSs (neoagarobiose, neoagarotetraose, neoagarohexaose) at 30°C for 4 h, respectively. The reaction products were analyzed by TLC.

### Genome Sequencing and Analyzing

Strain LGH was grown in LB medium and its genomic DNA was extracted by Universal Genomic DNA Extraction Kit (TaKaRa). The genomic DNA of strain LGH was constructed as Illumina PE library (∼300 bp) and Roche454 MP library (∼1 Kb) using the TruSeq™ DNA Sample Prep Kit and TruSeq PE Cluster Kit v3-cBot-HS. After filtering ineffective and low-quality data, the Illumina PE library contained 3,831,007 total high quality read pairs with 204-fold depth of coverage and the Roche454 MP library harbored 79,364 total reads with fourfold depth of coverage. The two libraries were assembled by 454’s GS *De Novo* Assembler (Newbler) v2.8, rRNA and tRNA genes were detected using Barrnap version 0.4.2 software and tRNAscan-SE version 1.3.1, respectively. The protein-coding sequences were predicted by Glimmer version 3.02 software using default parameters. The annotation of the predicted open reading frames was determined by using BLAST searches of non-redundant protein sequences from the NCBI, Swiss-Prot, COG, and KEGG databases.

### Quantitative Real Time-PCR Analysis

LGH was grown in MS and NAOS medium until the late exponential phase. Total RNA was extracted using a MiniBEST Universal RNA Extraction Kit (TaKaRa), and the concentration and purity of extracted RNA was measured by NonoDrop 2000 (Thermo Fisher Scientific). Then, approximately 0.5 μg RNA was added to synthesize cDNA using a PrimeScript™ RT reagent Kit (TaKaRa). TB Green *Premix Ex Taq*™ (TaKaRa) was used in qPCR reaction and the 96-well plate with resulting mixture was performed by Step One Plus Real Time PCR system (Applied Biosystems, Thermo Fisher Scientific). The values of 2^–ΔΔCt^ were calculated to estimate the differences of gene expression. ΔΔCt = (Ct_target gene_ – Ct_housekeeper gene_)_experimental group_ − (Ct_target gene_ – Ct_housekeeper gene_)_control group_. The primers used for qPCR are listed in Table 1.

**TABLE 1:** Primers used in this study.

Primers	Sequence (5′–3′)^a^
QrpoBF	CGAACGTGGGCCGCTATAAGGTCAA
QrpoBR	CTTCTCAAGCATCGGCAAGATTTGA
QagaWF	TTGAAGCGACTATTTGCAAGCCTGC
QagaWR	GCAGTGCAGCGTTAGAGCCTGTTAC
Q4969F	GACGGGGTGTTCCGGAACTGCTCCA
Q4969R	GCCGCCGCCTTCATACCACCAGCCT
Q4994F	CTTGCGGAATGATGAAGCGGGCCTG
Q4994R	TTGCCATCATGAATTTCCCATCGCT
Q4996F	CGCAATGGACAAGACTATTATCGTTT
Q4996R	CCTGCTGCGCCCGCTCGGACGATTC
Q5009F	CGGCTATACCTACACGGGCGGCGTA
Q5009R	AAGCAATGCCGCCGCGCCCTACATA
Q5012F	TATTGAAGGCCAATACCTGCTCAGG
Q5012R	GGTCAAACGATTATCGCCATCGCCG
Q5015F	AGGTATGGCCATGGGATTGGTCCGA
Q5015R	CTTGGAGCGTCTCAAATAGGGATGC
Q5037F	TCAATTCGGTTATGGAGGACGGCAT
Q5037R	CATGTAAACGGTGCTGTTCGACATC
Q5055F	ATGGCATGTCCTCCAAGCACTGGGC
Q5055R	CCAAGCCGCTGCTCCCGCATTCGAC
pETAgaWF (*Kpn*I)	GGGGTACCGCCACCCCGTTCCCTACTTTGAACT
pETAgaWR (*Xho*I)	CCGCTCGAGCTTTGATATTAGCAAATGATCCATT
pETCL4969F (*Bgl*II)	GAAGATCTGATGCGACAAGTATGGAGCTTAAAC
pETCL4969R (*Xho*I)	CCGCTCGAGGACACCGCCGTCGGCTGCATCTCTC
pETCL4994F (*Kpn*I)	CGGGTACCATGAGAGGAATTCCTTTTATACAGG
pETCL4994R (*Xho*I)	CCGCTCGAGTTCGACGACTTTAACCTTACAGGTA
pETCL4996F (*Kpn*I)	CGGGTACCATGATGAAGGGCAATCGCAATGGAC
pETCL4996R (*Xho*I)	CCGCTCGAGCTTATAATTAATGAAAACAGTTCGC
pETCL5009F (*Kpn*I)	CGGGTACCATGGCTAATGGAAAACGAATTACTC
pETCL5009R (*Xho*I)	CCGCTCGAGAAATAATTGCTTGTAAGGCTGAAGC
pETCL5012F (*Nde*I)	GGAATTCCATATGAAAAAAGAAAGCGCTGCAACGA
pETCL5012R (*Xho*I)	CCGCTCGAGATCCTTTTCTCGCTGCGCTTTCTTG
pETCL5015F (*Nde*I)	GGAATTCCATATGAGACAAGAAAGCTTGGCTATGC
pETCL5015R (*Xho*I)	CCGCTCGAGGTGATTGAACTTATTACTGCACAAA
pBADCL5015F (*Xho*I)	CCGCTCGAGATGAGACAAGAAAGCTTGGCTATGC
pBADCL5015R (*Kpn*I)	CGGGTACCATCTAGTGATTGAACTTATTACTGCA
pETCL5037F (*Sal*I)	ACGCGTCGACATGAAAGCGATGACGAATGAGGAAC
pETCL5037R (*Xho*I)	CCGCTCGAGCAAGAGACTAATTACAGTTTTACC
pETCL5055F (*Kpn*I)	CGGGTACCATGTCCTCCAAGCACTGGGCGGATG
pETCL5055R (*Sal*I)	ACGCGTCGACCTTCTGTGACAAACGAAGGTTATCG
pETAgWH2AF (*Sal*I)	ACGCGTCGACATGCCTTGTAAAACTTTACTTAATA
pETAgWH2AR (*Xho*I)	CCGCTCGAGGCCATGGTTCACCTCTAGCTGTTCG
pETAgWH117F (*Nde*I)	GGAATTCCATATGATGCTAAAATCAGCACGAAAGCTC
pETAgWH117R (*Xho*I)	CCGCTCGAGGTTGGTATTCTGGAAGGTACCAGCGT

*^a^Restriction sites are underlined.*

### Gene Cloning and Protein Purification

Eight candidate genes (*cl4969*, *cl4994*, *cl4996*, *cl5009*, *cl5012*, *cl5015*, *cl5037*, and *cl5055*) were amplified by PCR using genome of strain LGH as template. Two reported genes (*agawh117* and *agawh2a*) were amplified by PCR using genome of *A. gilvus* WH0801 as template. The primers used for cloning are listed in Table 1. PCR products were digested by restriction enzymes. The digested products were ligated into pET29a and then the resulting plasmids were transformed into *E. coli* BL21 (DE3) pLysS cells. Clones were screened by 50 μg ml^–1^ kanamycin and plasmid sequencing and *cl5015* was also cloned into pBAD-HisA. The recombinant plasmid was transformed into *E. coli* TOP10 and then screened by 100 μg ml^–1^ ampicillin.

The confirmed recombinant strains were grown at 37°C in LB medium with 50 μg ml^–1^ of kanamycin until OD_600_ value reached to 0.5. 1 mM isopropyl-β-D-thiogalactopyranoside (IPTG) was added to induce the gene expression at 16°C for 16 h. The overexpression of pBAD-CL5015 was induced by 1 mM L-arabinose. Cells were harvested and resuspended in 10 mM Tris-HCl buffer (pH 7.0) and then lysed by sonication. Centrifugation at 15,000 × g for 30 min was performed to remove cell debris. Supernatant was purified by a 2-ml volume of NTA-Ni^2+^ column (Sangon) at 4°C. The binding buffer contained 300 mM NaCl, 50 mM sodium phosphate, 10 mM imidazole, 10 mM Tris base (pH 8.0), and the elution buffer was composed of 300 mM NaCl, 50 mM sodium phosphate, 250 mM imidazole, and 10 mM Tris base (pH 8.0). The purified enzymes were desalted, concentrated with a Millipore Amicon ultra-15 centrifugal filter unit, and stored in 10 mM potassium phosphate buffer (pH 7.0). The concentration of recombinant enzyme was quantified by a protein assay kit with bovine serum albumin as a standard.

### Preparation of Agarotetraose

Approximately 0.5 ml of 5 mg ml^–1^ α-agarase AgaWS5 was incubated with 1.5 ml of 1.5% agarose at 40°C in 1 mM potassium phosphate buffer (pH 7.0) for 12 h. The reaction mixture was cooled at 4°C for 10 min, was centrifuged at 15,000 × *g* for 30 min, and then the supernatant was filtered with a Millipore Amicon ultra-2 centrifugal filter unit for protein removal. The filtered liquid containing agarotetraose was collected and stored in −20°C. The agarotetraose was then identified by TLC and mass analysis.

### Enzymatic Conversion Assays

To determine the depolymerization of agarose by CL5055, 50 μl of 2 mg ml^–1^ CL5055 was mixed with 350 μl of 0.5% agarose at 40°C ranging from 0 to 1,440 min. The products were determined by TLC and mass spectrometry. To further determine the depolymerization of NAOSs by CL5055, neoagarobiose, neoagarotetraose, and neoagarohexaose (10 μl of 2.5 mg ml^–1^) were mixed with 10 μl of 2 mg ml^–1^ CL5055 at 40°C for 2 h, respectively. The products were then determined by TLC and mass spectrometry. The hydrolysis activity of CL5055 was also quantified by the 3,5-dinitrosalicylic acid (DNS) method, which had been described previously (Li et al., 2015). Briefly, the reaction mixture was mixed with an equal amount of DNS reagent solution, heated in a boiling water bath for 5 min, and then the reducing sugar of the mixture was measured spectrophotometrically at 540 nm.

In the ABG-mediated depolymerization assays, agarotriose was used as substrate. CL4969, CL4994, CL5037, AgWH2A (10 μl of 2 mg ml^–1^) were incubated with 10 μl of 2.5 mg ml^–1^ agarotriose at 30°C for 4 h, respectively. In the assays, all the enzymatic reactions were incubated in 10 mM potassium phosphate buffer (pH 7.5). The products were then measured by high performance liquid chromatography (HPLC).

In the α-NAOS hydrolase-mediated depolymerization assays, neoagarobiose and neoagarotetraose were used as substrates, respectively. CL5012, CL5015, AgaWH117 (10 μl of 2 mg ml^–1^) were mixed with 10 μl of 2.5 mg ml^–1^ neoagarobiose or neoagarotetraose at 30°C for 4 h, respectively. The products were measured by high performance liquid chromatography-mass spectrometry (HPLC-MS).

To determine the depolymerization ability of CL5012 and CL4994, both proteins (10 μl of 2 mg ml^–1^) were incubated with 5 μl of 2.5 mg ml^–1^ agarotetraose solution at 30°C for 2 h, respectively. CL5012 was further incubated with the supernatant of the reaction product of CL4994 at 30°C for 2 h. All products were measured by HPLC-MS.

To determine the conversion ability of CL4996 to L-AHG, 20 μl of 2 mg ml^–1^ CL4996 was mixed with 80 μl of 2.5 mg ml^–1^ L-AHG with 1 mM NADP^+^ at 30°C for 2 h. In the assay, VejAHGD was also used as a positive control. The products were measured by GC-MS.

To determine the conversion ability of CL5009 to AHGA, CL4996 was incubated with L-AHG at 30°C for 2 h as described above, and then 20 μl of 2 mg ml^–1^ CL5009 was added into the reaction. The reaction was continuously incubated at 30°C for 2 h. In the assay, VejACI was also used as a positive control. The products were measured by GC-MS.

### Analysis of Enzymatic Products

For TLC analysis, reaction products were applied to silica gel 60 TLC plate (Merck, Germany), and then developed by *n-*butanol-ethanol-water solution (3:3:1, by volume). Spots were visualized by using 10% (by volume) H_2_SO_4_ in ethanol and heating at 105°C for 10 min. In addition, the developing solvent for agarotetraose was *n-*butanol-ethanol-water solution (5:4:2, by volume).

For mass spectrometry analysis, the spots of TLC plate corresponding to the hydrolyzed products were scraped out and dissolved in 80% acetonitrile for drying. The molecular mass distribution of the products was determined using an Agilent 6460 triple-quadrupole mass spectrometer equipped with electrospray ionization (ESI) under negative-ion ionization conditions. The ESI-MS conditions were as follows: all injections were 5 μl; drying gas temperature was at 350°C; drying gas flow (nitrogen) was at 10 L min^–1^; nebulizer gas pressure (nitrogen) was at 30 psi; and capillary voltage was at 4,000 V. Negative ions were acquired in full scan mode in the range of m/z 100–1,000 molecular mass units for identification within a 1^–s^ scan time interval.

For HPLC analysis, the reaction products were determined by Agilent Infinity 1260 (Agilent Technologies) equipped with an evaporative light scattering detector G4260B (ELSD, Agilent Technologies). The chromatographic column was Alltech Prevail Carbohydrate ES column (250 × 4.6 mm). The HPLC conditions were as follows: all injections were 10 μl mobile phase, acetonitrile-water (8:2 by volume) had a flow rate of chromatography of 1 ml min^–1^; column temperature was 30°C; detector temperature was 30°C; and gas flow was 1.6 L min^–^

For HPLC-MS analysis, the reaction products were determined by Agilent high-performance liquid chromatography triple-quadrupole mass spectrometer (LC-1290 MS-6460, Agilent Technologies) equipped with ESI under negative-ion ionization conditions. The chromatographic column was Alltech Prevail Carbohydrate ES column (250 × 4.6 mm). The HPLC-MS conditions were as follows: all injections were 10 μl mobile phase, acetonitrile-water (8:2 by volume) had a flow rate of chromatography of 0.75 ml min^–1^; column temperature was 30°C; drying gas temperature was 350°C; and capillary voltage of mass was 4 kV. Negative ions were acquired and mass spectra were in the range of 100–1,000 m/z.

For GC-MS analysis, the reaction mixtures were centrifuged at 15,000 × g for 30 min, then the supernatant was collected and lyophilized. The dried samples were derivatized by adding 50 μl of 20 mg ml^–1^ methoxyamine hydrochloride dissolved in pyridine at 75°C for 30 min, then 60 μl of N-methyl-N-(trimethylsilyl) trifluoroacetamide was added to each sample and incubated at 37°C for 30 min. After centrifuging at 15,000 × g for 30 min, supernatant was collected for analysis. Bruker 450-GC 320-MS system (Bruker Daltonics Inc.), equipped with a DB5-MS column (30 m × 0.25 mm ID, 0.25-μm film thickness), was used to analyze reaction products. The temperature program was as follows: all injections were 1 μl and the initial temperature was 100°C for 2 min, then was increased to 230°C for 15°C min^–1^, and maintained at 230°C for 20 min. The temperature was then increased to 300°C at 20°C min^–1^, and this temperature was maintained for 10 min. Electron ionization was performed at 70 eV, and the temperature of the ion source and transfer line was 230°C. Mass spectra was in the range of 50–500 m/z.

### Biochemical Properties of CL5055, CL4994, and CL5012

To determine the optimal pH for each enzyme, the enzymatic reactions were performed in different conditions. CL5055 was determined at 45°C in a pH range from 3.0 to 12.0 at an interval of 1; CL4994 was determined at 30°C in a pH range from 4.0 to 10.0 at an interval of 1; CL5012 was determined at 35°C in a pH range from 4.0 to 10.0 at an interval of 1. The pH stability was determined after preincubation of enzymes in different buffers for 1 h. Meanwhile, the preincubation temperatures depended on enzymes, CL5055 at 45°C, CL4994 at 30°C, and CL5012 at 35°C. Buffers used in this section were: 10 mM Na_2_HPO_4_-citrate buffer of pH 3.0–5.0, 10 mM potassium phosphate buffer of pH 6.0–7.0, 10 mM Tris-HCl buffer of pH 8.0–9.0, and a 10 mM glycine-NaOH buffer of pH 10.0–12.0.

The optimal temperatures of each enzyme were determined in different conditions. CL5055 was determined in 10 mM Tris-HCl buffer (pH 9.0) at a temperature range from 30 to 65°C at an interval of 5; CL4994 was determined in 10 mM potassium phosphate buffer (pH 7.0) at a temperature range from 20 to 45°C at an interval of 5; CL5012 was determined in 10 mM Tris-HCl buffer (pH 8.0) at a temperature range from 20 to 50°C at an interval of 5. The temperature stability was determined after preincubation of enzymes at different temperatures for 1 h. The preincubation pH depended on enzymes, CL5055 at pH 9.0, CL4994 at pH 7.0, and CL5012 at pH 8.0.

The effects of potential inhibitors or activators on the enzyme activity were determined by adding various metal ions and chemical reagents at a final concentration of 10 mM. The relative activity was defined as a percentage of the activity obtained in the absence of an additive. Three independent determinations were performed for all above measurements in this section.

### Kinetic Parameters of CL5055, CL4994, and CL5012

To determine the kinetic parameters of each enzyme, approximately 50 μg ml^–1^ CL5055 was incubated with agarose at a final concentration of 0.5–10 mg ml^–1^ in 10 mM Tris-HCl buffer (pH 9.0) at 45°C for 10 min. One activity unit (U) of CL5055 was defined as the amount of enzyme required to produce 1 μmol of reducing sugar per minute at 45°C. Approximately 25 μg ml^–1^ CL4994 was incubated with agarotriose at a final concentration of 0.5–7.5 mg ml^–1^ in the 10 mM potassium phosphate buffer (pH 7.0) at 30°C for 10 min. Owing to the incomplete separation of D-galactose and neoagarobiose (Supplementary Figure 1), one U of CL4994 was defined as the amount of enzyme required to consume 1 μmol of agarotriose per minute at 30°C. Approximately 100 μg ml^–1^ CL5012 was incubated with neoagarobiose at a final concentration of 0.5–7.5 mg ml^–1^ in 10 mM Tris-HCl buffer (pH 8.0) at 35°C for 10 min. One U of CL5012 was defined as the amount of enzyme required to produce 1 μmol of L-AHG per minute at 35°C. Kinetic parameters were calculated from non-linear regression data analysis against various substrate concentrations using origin 8.0 and three independent determinations were performed.

### Measurement of Viscosity and Substrate Specificity of the CL5055

To determine the kinematic viscosity of the CL5055, the Ubbelohde viscometer was used. 20 μl of 2 mg ml^–1^ CL5055 was mixed in 15 ml of 10 mM potassium phosphate buffer (pH 8.0) with 0.5% agarose as substrate. The reaction was incubated at 45°C at various reaction times ranging from 0 to 120 min and the efflux time was measured at each time. The experiment was repeated three times.

The substrate specificity of the CL5055 was measured by using two artificial chromogenic substrates, *p*-nitrophenyl-α-D-galactopyranoside and *p*-nitrophenyl-β-D-galactopyranoside, respectively. Twenty microliter of 2 mg ml^–1^ CL5055 was incubated in 700 μl of 10 mM potassium phosphate buffer (pH 8.0) with each substrate at a final concentration of 3 mg ml^–1^ at 45°C for 2 h. The reaction was stopped by addition of 500 μl of 1 M Na_2_CO_3_. The activity of the enzyme was measured spectrophotometrically at 420 nm by evaluating the release of p-nitrophenol from the hydrolysis of the artificial chromogenic substrate. Three independent determinations were performed.

Accession numbers of enzymes involved in this study, NCBI accession numbers: CL4969, **QTH40778.1**; CL4994, **QTH40796.1**; CL4996, **QTH40798.1**; CL5009, **QTH40807.1**; CL5012, **QTH40809.1**; CL5015, **QTH40812.1**; CL5037, **QTH40833.1**; CL5055, **QTH40851.1**.

## Results

### Sequencing of *Cohnella* sp. LGH Genome for Screening the Candidate Genes Involved in the Agarose Catabolism Pathway

**FIGURE 1:**
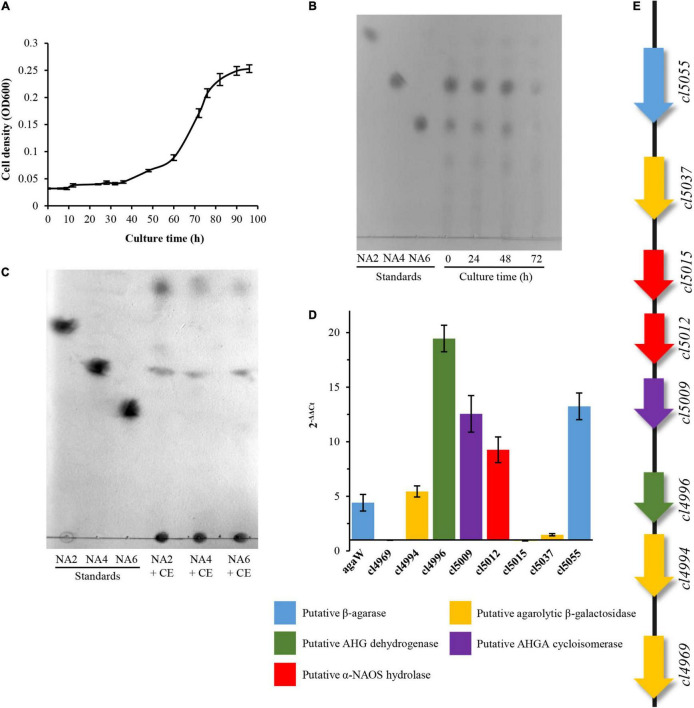
Utilization of NAOSs by *Cohnella* sp. LGH and qPCR analysis of agarolytic candidate genes. **(A)** Growth curve of strain LGH cultured by NAOS medium. **(B)** TLC analysis of residual NAOSs from liquid medium. **(C)** TLC analysis of crude enzyme incubated with neoagarobiose, neoagarotetraose, and neoagarohexaose. **(D)** qPCR analysis of relative quantification of eight candidate genes involved in agarolytic pathway. The mean values and standard deviations were calculated by three replicate experiments. **(E)** Cluster of putative functional genes involving in agarose catabolism of strain LGH. AHG, 3,6-anhydro-L-galactose; AHGA, 3,6-anhydro-galactonate; CE, crude enzyme; NA2, neoagarobiose; NA4, neoagarotetraose; NA6, neoagarohexaose; NAOS, and neoagarooligosaccharide.

Previously, we found that *Cohnella* sp. LGH was capable of utilizing agarose as a sole carbon source for growth and showed strong agarolytic activity. One endo-type β-agarase AgaW was responsible for the hydrolysis of agarose into the major product of neoagarotetraose and has been identified and characterized from strain LGH (Li et al., 2015). Here, this bacterium, as expected, was capable of utilizing various α-NAOSs including neoagarotetraose as a sole carbon source for growth and its crude enzyme extract also showed a strong α-NAOSs hydrolytic activity (Figures 1A,B). Thin layer chromatography (TLC) did not detect any obvious end product signals in the α-NAOSs metabolites (Figure 1C). These results suggest that there might be a α-NAOSs associated catabolism pathway in strain LGH.

In order to investigate the possible pathway, we then sequenced and analyzed the complete genome of strain LGH. The draft genome sequence of *Cohnella* sp. LGH was deposited in GenBank under the accession number CP072117. The draft assembly of *Cohnella* sp. LGH displayed a single closed contig sequence of 8,896,274 bases (Supplementary Figure 2), with 53.73% G + C content, including 25 rRNA, 67 tRNA, and 7,357 open reading frames (ORFs). Combined to the annotation analysis of Non-redundant database and Swiss-Prot database, 6,836 proteins were functionally annotated. Based on comparative sequence analysis and protein functional domain analysis, eight candidate genes that may be involved in the agarose catabolism pathway were screened and are shown in Figure 1E, including *cl5055* encoding a putative β-agarase; *cl5012* and *cl5015* encoding putative α-NAOS hydrolases; *cl4969*, *cl4994*, and *cl5037* encoding putative ABGs; *cl4996* encoding a putative L-AHG dehydrogenase; *cl5009* encoding a putative 3,6-anhydrogalactonate cycloisomerase. Quantitative real-time polymerase chain reaction (qPCR) analysis further showed that the transcriptional levels of five candidate genes, containing *cl4994*, *cl4996*, *cl5009*, *cl5012*, and *cl5055*, increased when strain LGH utilized α-NAOSs as a sole carbon source for growth (Figure 1D).

### Depolymerization of Neoagarobiose and Neoagarotetraose by the α-Neoagarooligosaccharides Hydrolase CL5012

**FIGURE 2:**
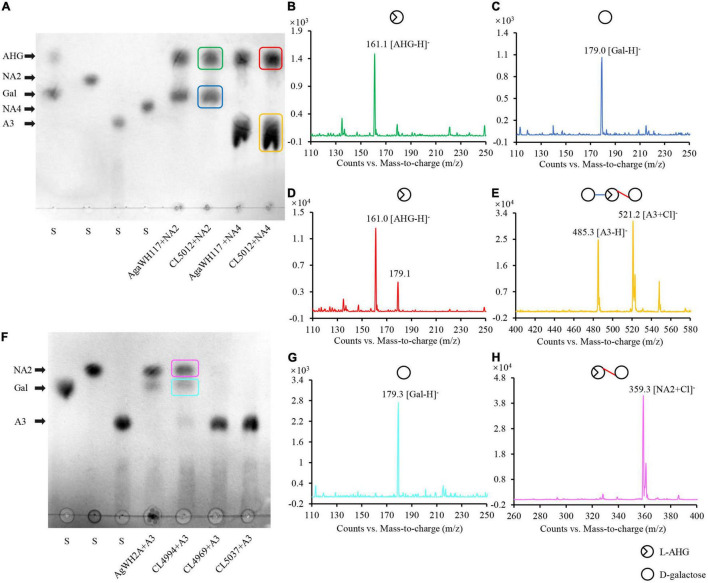
Analysis of the reaction products of α-NAOS hydrolase CL5012 and agarolytic β-galactosidase CL4994. **(A)** TLC analysis of the hydrolysis products of CL5012 incubated with neoagarobiose and neoagarotetraose, respectively. **(B)** Mass spectrum of one spot corresponding to L-AHG. **(C)** Mass spectrum of one spot corresponding to D-galactose. **(D)** Mass spectrum of one spot corresponding to L-AHG. **(E)** Mass spectrum of one spot corresponding to agarotriose. **(F)** TLC analysis of the hydrolysis products of CL4969, CL4994, and CL5037 incubated with agarotriose, respectively. **(G)** Mass spectrum of one spot corresponding to D-galactose. **(H)** Mass spectrum of one spot corresponding to neoagarobiose. AHG, 3,6-anhydro-L-galactose; A3, agarotriose; Gal, D-galactose; NA2, neoagarobiose; NA4, neoagarotetraose; S, standard substance.

The endo-type β-agarase AgaW depolymerized agarose into two α-NAOS products, neoagarotetraose as the major end product and neoagarobiose as the minor end product (Li et al., 2015). Both end α-NAOS products could be further degraded in strain LGH as described above. After comparative sequence analysis and protein functional domain analysis, two candidate α-NAOS hydrolases CL5012 and CL5015, responsible for the end products degradation, were screened from the LGH genome. Both of them not only shared approximately 50% amino acid sequence identity to two α-NAOS hydrolases ScJC117 (accession NO. CAB61805) from *S. Coelicolor* A3(2) (Jiang et al., 2020b) and SdNABH (accession NO. ABD81917) from *S. degradans* 2-40^T^ (Ha et al., 2011), but also harbored the conserved GH117 family domains (accession NO. CD08992) (Supplementary Figure 3) which were widely present in all reported α-NAOS hydrolases (Jiang et al., 2020a). We then overexpressed CL5012 and CL5015 in *E. coli* BL21 and purified both of them. After incubation of neoagarobiose with CL5012, TLC detected two spots which shared the same traveling distance as the depolymerization products of neoagarobiose by the reported α-NAOS hydrolase AgaWH117 from *A. gilvus* WH0801 (Figure 2A). One spot had a strong quasimolecule ion at *m/z* of 161.1, represented [M–H]^–^, corresponding to L-AHG (Figure 2B). Another spot had a strong quasimolecule ion at *m/z* of 179.0, represented [M–H]^–^, corresponding to D-galactose (Figure 2C). CL5012 also depolymerized neoagarotetraose like AgaWH117 into two products as shown in TLC analysis (Figure 2A). One product had a strong quasimolecule ion at *m/z* of 161.0, represented [M–H]^–^, corresponding to L-AHG (Figure 2D). Another product had a same quasimolecule ion at *m/z* of 485.3 and 521.2, represented [M–H]^–^ and [M + Cl]^–^, corresponding to agarotriose (Figure 2E). The combined results demonstrated that CL5012 had α-NAOS hydrolase activity. On the other hand, another candidate protein CL5015 did not show any α-NAOS hydrolase activity since it degraded neither neoagarobiose nor neoagarotetraose (Supplementary Figure 4).

We then detected the enzymatic properties of CL5012 by utilizing neoagarobiose as the substrate. CL5012 showed the maximum activity at pH 8.0, and its relative activity dropped quickly at pH beyond 6.0 and 9.0. It was stable at a pH range from 6.0 to 8.0 and almost lost activity at pH 4.0 after being pre-incubated for 60 min (Supplementary Figure 5A). The protein showed enzymatic activity at temperatures ranging from 20 to 50°C, with the maximum activity at 35°C, and its relative activity dropped dramatically at temperature beyond 35°C. CL5012 exhibited more than 80% of initial activity at temperatures ranging from 20 to 35°C for 60 min pre-incubation, and its stability dropped, obviously, at temperature beyond 35°C (Supplementary Figure 5B). Its enzymatic activity was completely inhibited by metal-chelator EDTA and surfactant SDS and partially inhibited by Mg^2+^, Ca^2+^, and Mn^2+^. On the contrary, Zn^2+^ and Cu^2+^ could obviously increase its enzymatic activity, suggesting that Zn^2+^ or Cu^2+^ might be involved in the catalytic reaction of CL5012 (Supplementary Table 1). According to the amino acid composition analysis, theoretical molecular weight of CL5012 was 41.90 kDa. The *K*_m_, *V*_max_, *k*_cat_, and *k*_cat_/*K*_m_ values of CL5012 for neoagarobiose as substrate were 4.45 mg ml^–1^ (M), 70.66 U mg^–1^, 43.5 s^–1^, and 4.8 × 10^3^ M^––1^ s^––1^, respectively. According to the classification analysis of dbCAN2 server,^1^ CL5012 was further classified into GH117 family.

### Depolymerization of Agarotriose by the Agarolytic β-Galactosidase CL4994

As described above, CL5012 hydrolyzed neoagarotetraose into L-AHG and agarotriose. TLC analysis further showed that the crude enzyme extract of strain LGH could continue to degrade agarotriose, but the identified protein involved in the agarose catabolism in LGH, as the β-agarase AgaW and the α-NAOS hydrolase CL5012, did not show any agarotriose degradation activity (data not shown). The results suggested there should be other protein responsible for the agarotriose degradation in LGH. We then utilized two reported agarotriose hydrolase sequences belonging to the family of β-galactosidase like protein, the ABG VejABG (accession NO. WP_014232195) from *Vibrio* sp. EJY3 (Lee et al., 2014) and the ABG AgWH2A (accession NO. MG456856) from *A. gilvus* WH0801 (Yang et al., 2018), as the query sequences to retrieve the LGH genome. Three proteins CL4969, CL4994, and CL5037 harboring the conserved LacZ domain (accession NO. COG3250) of the β-galactosidase like protein family (Supplementary Figure 3) and only sharing the highest sequence identity of 21 ∼ 44% to both query sequences were selected as the candidate proteins. We then overexpressed and purified these candidate proteins. After incubation of agarotriose with three candidate proteins, respectively, TLC detected two spots in the reaction of agarotriose with CL4994. Both spots shared the same traveling distance as the depolymerization products of agarotriose by the reported ABG AgWH2A (Figure 2F). One spot had a strong quasimolecule ion at *m/z* of 179.3, represented [M–H]^–^, corresponding to D-galactose (Figure 2G). Another spot had a strong quasimolecule ion at *m/z* of 359.3, represented [M + Cl]^–^, corresponding to neoagarobiose (Figure 2H). Thus, according to the results, CL4994 was identified as an ABG. In this assay, CL4969 and CL5037 did not show any agarotriose degradation activity.

Agarotriose was further utilized as the substrate to determine the enzymatic properties of CL4994. CL4994 exhibited maximum activity at pH 7 and retained more than 80% of maximum activity at a pH range from 5.0 to 8.0. CL4994 was stable at a pH range from 6.0 to 8.0, retaining at least 80% of initial activity for 60 min pre-incubation (Supplementary Figure 5C). The optimal temperature of CL4994 was 30°C, and retained more than 90% of maximum activity temperature ranging from 20 to 35°C. CL4994 retained at least 90% of initial activity at temperature ranging from 20 to 35°C for 60 min pre-incubation, and its relative activity dropped obviously at temperatures beyond 35°C (Supplementary Figure 5D). The enzymatic activity of CL4994 was completely inhibited by Cu^2+^ and surfactant SDS and partially inhibited by Ca^2+^, Fe^2+^, and Fe^3+^. No significant activation or inhibition of CL4994 was observed by Na^+^, K^+^, Mg^2+^, Mn^2+^, Zn^2+^, and EDTA (Supplementary Table 2). According to the amino acid composition analysis, theoretical molecular weight of CL4994 was 93.62 kDa. The *K*_*m*_, *V*_*max*_, *k*_*cat*_, and *k*_*cat*_/*K*_*m*_ values of CL4994 for agarotriose as substrate were 3.90 mg ml^–1^ (M), 79.41 U mg^–1^, 52.9 s^–1^, and 4.4 × 10^3^ M^–1^s^–1^, respectively. The classification analysis of dbCAN2 server showed that CL4994 was belong to GH2 family.

### Depolymerization of Agarotetraose by CL4994 and CL5012

**FIGURE 3:**
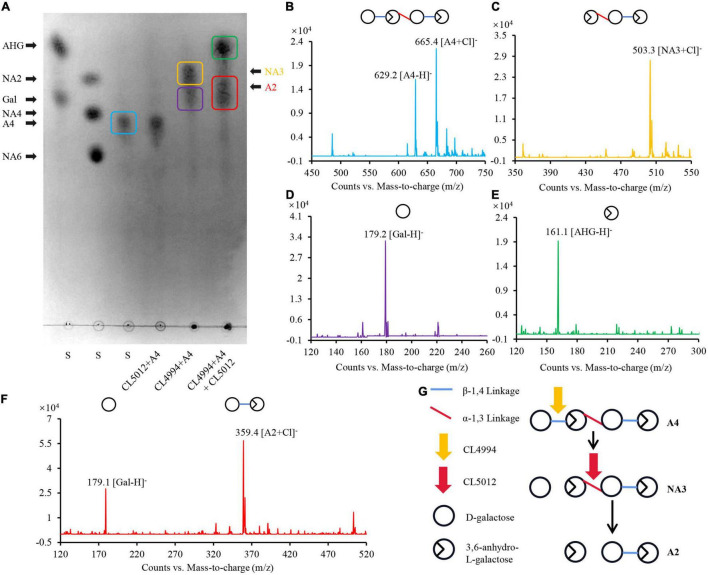
Analysis of depolymerization process of agarotetraose by agarolytic β-galactosidase CL4994 and α-NAOS hydrolase CL5012. **(A)** TLC analysis of hydrolysis products of CL5012, CL4994 incubated with agarotetraose, respectively. **(B)** Mass spectrum of one spot corresponding to agarotetraose. **(C)** Mass spectrum of one spot corresponding to neoagarotriose. **(D)** Mass spectrum of one spot corresponding to D-galactose. **(E)** Mass spectrum of one spot corresponding to L-AHG. **(F)** Mass spectrum of two spots corresponding to agarobiose and D-galactose. **(G)** Schematic model of depolymerization of agarotetraose by CL4994 and CL5012. AHG, 3,6-anhydro-L-galactose; A2, agarobiose; A4, agarotetraose; Gal, D-galactose; NA3, neoagarotriose; S, standard substance.

Besides β-agarases, bacterial α-agarases were also capable of hydrolyzing agarose into the major end product agarotetraose, such as AgaWS5 from *Catenovulum sediminis* WS1-A and AgaD from *Thalassomonas* sp. LD5 (Zhang et al., 2018; Lee et al., 2019). Agarotetraose was usually considered to be further depolymerized into agarotriose and L-AHG by chemical treatment (Zhang et al., 2018), but its depolymerization in bacteria still remain unclear. We have demonstrated above, that the combined action of CL5012 and CL4994 depolymerized neoagarotetraose into monosaccharides. Here, we also found that the combined action of CL5012 and CL4994 also play a critical role in the depolymerization of agarotetraose despite the fact that α-agarase has not been identified in strain LGH. In the assays, the substrate agarotetraose came from the hydrolysis of agarose by the reported α-agarase AgaWS5 (Lee et al., 2019). It was also identified by TLC analysis (Figure 3A) and mass spectra (Figure 3B). After incubation of agarotetraose with CL5012 or CL4994, respectively, TLC detected two spots in the reaction of agarotetraose with CL4994 (Figure 3A). One spot had quasimolecule ion at *m/z* of 503.3, represented [M + Cl]^–^, corresponding to neoagarotriose (Figure 3C). Another spot had a strong quasimolecule ion at *m/z* of 179.2, represented [M–H]^–^, corresponding to D-galactose (Figure 3D). CL5012 did not show any depolymerization activity to agarotetraose. However, after incubation of CL5012 with the hydrolysis products of agarotetraose by CL4994, the spot corresponding to neoagarotriose disappeared and three spots were finally present in the TLC plate. One spot had a strong quasimolecule ion at *m/z* of 161.1, represented [M–H]^–^, corresponding to L-AHG (Figure 3E). The other two spots were too close to separate. Thus, we scraped them from the TLC plate and then identified them together by mass spectra. The results showed a strong quasimolecule ion at *m/z* of 179.1 and 359.4, represented [M–H]^–^ and [M + Cl]^–^, corresponding to D-galactose and agarobiose, respectively (Figure 3F). The combined result indicated that agarotetraose was depolymerized by CL4994 into D-galactose and neoagarotriose, and then neoagarotriose could be further depolymerized by CL5012 into L-AHG and agarobiose (Figure 3G).

### Depolymerization of Agarose to Agaro-Oligosaccharides by the β-Agarase CL5055

**FIGURE 4:**
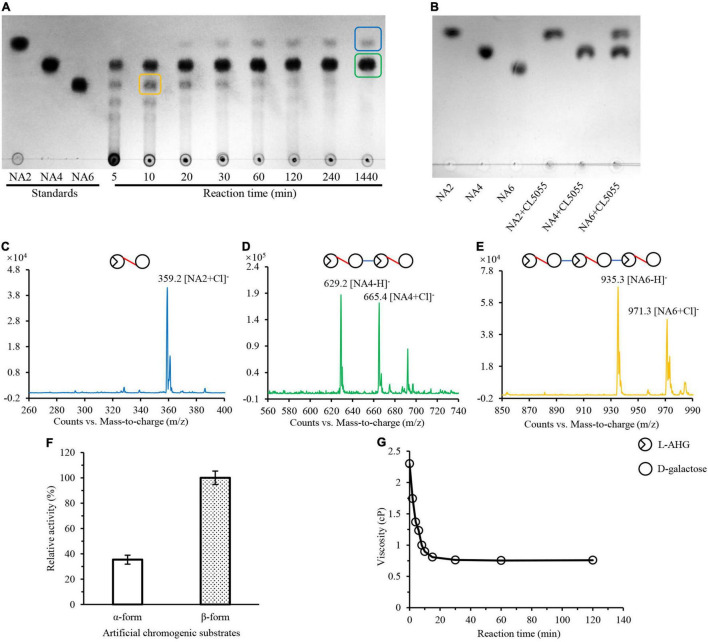
Analysis of reaction products and mode of action of CL5055. **(A)** TLC analysis of the hydrolysis products of CL5055 incubated with agarose at the indicated intervals. **(B)** CL5055 hydrolyzed neoagarohexaose into neoagarobiose and neoagarotetraose as two end products. **(C)** Mass spectrum of one spot corresponding to neoagarobiose. **(D)** Mass spectrum of one spot corresponding to neoagarotetraose. **(E)** Mass spectrum of one spot corresponding to neoagarohexaose. **(F)** Substrate specificity of CL5055 toward two artificial chromogenic substrates, p-nitrophenyl-α-D-galactopyranoside and p-nitrophenyl-β-D-galactopyranoside. **(G)** Viscosity changes of the CL5055 reaction mixture. AHG, 3,6-anhydro-L-galactose; NA2, neoagarobiose; NA4, neoagarotetraose; NA6, neoagarohexaose.

Previously, we have reported an endo-type β-agarase AgaW in strain LGH. In this study, we found that another protein encoded by the gene *cl5055* in LGH genome might also be responsible for the hydrolysis of agarose since two conserved agarase CBM like domains (accession NO. pfam17992) were present in its sequence (Supplementary Figure 3). This protein with a potential 24 amino acid residues signal peptide only shared 51.0% sequence identity to the endo-type β-agarase AgaW (accession NO. KR296705) in strain LGH (Li et al., 2015), and did not obviously show overall sequence identity to any other characterized agarase. To further investigate the possible agarolytic activity of CL5055, we overexpressed and purified CL5055. After incubation of agarose with CL5055, the agarose hydrolytic products were separated and detected by TLC with the standard short-chain oligosaccharides, neoagarohexaose, neoagarotetraose, and neoagarobiose as controls. One spot, sharing the same traveling distance as neoagarohexaose, was obviously decreased after 30 min, and finally disappeared (Figure 4A). This intermediate product had a weak quasimolecule ion at *m/z* of 935.3, represented [M–H]^–^ and a strong quasimolecule ion at *m/z* of 971.3, represented [M + Cl]^–^, corresponding to neoagarohexaose (Figure 4E). Only two spots were detected from the reaction products after a long incubation time of 1,440 min (Figure 4A). One spot, sharing the same traveling distance as neoagarobiose, had a strong quasimolecule ion at *m/z* of 359.2, represented [M + Cl]^–^, corresponding to neoagarobiose (Figure 4C). Another spot, sharing the same traveling distance as neoagarotetraose, had two strong quasimolecule ions at *m/z* of 629.2 and *m/z* of 665.4, represented [M–H]^–^ and [M + Cl]^–^, respectively, corresponding to neoagarotetraose (Figure 4D). When we incubated excess CL5055 with the standard neoagarobiose, neoagarotetraose, and neoagarohexaose as substrates, respectively, neoagarobiose and neoagarotetraose were not hydrolyzed as expected, while neoagarohexaose was completely hydrolyzed into neoagarobiose and neoagarotetraose (Figure 4B). These results clearly demonstrated that CL5055 hydrolyzed agarose into neoagarotetraose and neoagarobiose as two end products through other short-chain oligosaccharides intermediates, such as neoagarohexaose. We also utilized *p*-nitrophenyl-α-D-galactopyranoside and *p*-nitrophenyl-β-D-galactopyranoside as the specific substrates to determine the mode of catalysis action of CL5055. CL5055 exhibited a strong hydrolytic activity toward *p*-nitrophenyl-β-D-galactopyranoside and showed a weak hydrolytic activity toward *p*-nitrophenyl-α-D-galactopyranoside (Figure 4F), indicating that CL5055 recognized and cleaved β-linkage. To investigate the hydrolysis pattern of CL5055, we then measured the change in viscosity of agarose during the enzymatic reaction. The result showed that viscosity dropped rapidly at the beginning time for 10 min and decreased gradually from 10 to 30 min (Figure 4G), suggesting that the cleavage type of CL5055 was endo type. Therefore, CL5055 was identified as an endo-type β-agarase.

The agarolytic activity of CL5055 was then measured by the 3,5-DNS method as previously described. CL5055 exhibited the maximum enzymatic activity at pH 9.0, and retained more than 80% of maximum activity at a pH range from 7.0 to 10.0. Meanwhile, the relative activity dropped obviously when pH was below 7.0, suggesting that CL5055 might be an alkaline agarase. CL5055 was stable at a pH range from 7.0 to 10.0 after being pre-incubated for 60 min (Supplementary Figure 5E). The optimal temperature of CL5055 was 45°C, and retained more than 80% of maximum activity ranging from 40 to 50°C. CL5055 exhibited thermostability from 30 to 45°C when it was pre-incubated for 60 min (Supplementary Figure 5F). The enzymatic activity of CL5055 was completely inhibited by Cu^2+^ and surfactant SDS, and partially inhibited by Mg^2+^, Zn^2+^, Ca^2+^, Mn^2+^, Ba^2+^, Fe^3+^, and EDTA. On the contrary, 10 mM DTT could increase enzymatic activity of CL5055 (Supplementary Table 3). According to the amino acid composition analysis, theoretical molecular weight of CL5055 was 113.51 kDa (removing signal peptide). The *K*_*m*_, *V*_*max*_, *k*_*cat*_, and *k*_*cat*_/*K*_*m*_ values of CL5055 for agarose as substrate were 4.42 mg ml^–1^ (M), 186.43 U mg^–1^, 8.6 × 10^3^s^–1^, and 1.9 × 10^7^ M^–1^s^–1^, respectively. Combined to its protein sequence phylogenetic tree analysis (Supplementary Figure 6) and classification analysis of dbCAN2 server, CL5055 was further classified into GH50 family.

## Discussion

**FIGURE 5:**
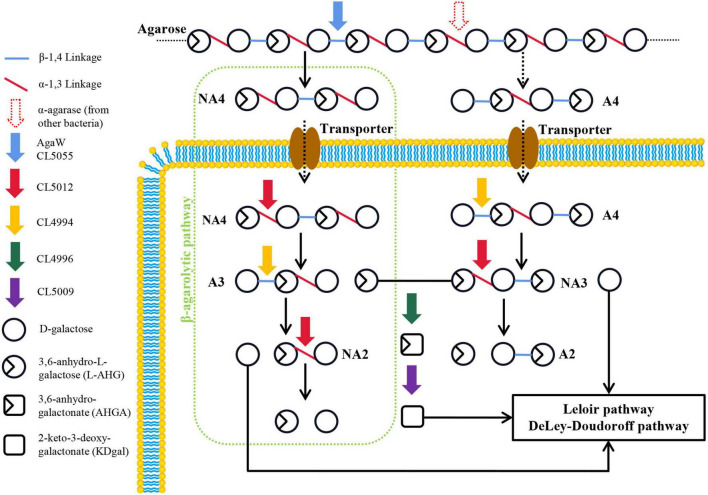
Schematic model of proposed agarolytic pathway in *Cohnella* sp. LGH. Extracellular β-agarases AgaW and CL5055 hydrolyzed agarose into neoagarotetraose, and meanwhile the α-agarase from other bacteria might hydrolyze agarose into agarotetraose. Two oligosaccharides were transported into cells via putative transporter. The neoagarotetraose was hydrolyzed into D-galactose and L-AHG by the combined actions of α-NAOS hydrolase CL5012 and agarolytic β-galactosidase CL4994. Meanwhile, D-galactose and L-AHG were released from agarotetraose by sequential actions of CL4994 and CL5012. D-galactose was metabolized through Leloir or DD pathway. L-AHG was converted into KDgal by the sequential actions of CL4996 and CL5009, and then KDgal was further metabolized by DD pathway. A2, agarobiose; A3, agarotriose; A4, agarotetraose; NA2, neoagarobiose; NA3, neoagarotriose; NA4, neoagarotetraose.

In this study, we mainly determined the catalytic function of a novel α-NAOS hydrolase CL5012 and a novel ABG CL4994 in the depolymerization of neoagarotetraose and agarotetraose in the terrestrial agar-degrading bacterium *Cohnella* sp. LGH by *in vitro* reactions. Based on the combined results, we proposed an Aux I type agarolytic pathway in strain LGH (Figure 5). A novel β-agarase CL5055 and a previously reported β-agarase AgaW (with putative signal peptides and likely being secreted out of cells) hydrolyzed agarose into neoagarotetraose. Neoagarotetraose might then be transported into cells via putative transporters. The intracellular α-NAOS hydrolase CL5012 depolymerized neoagarotetraose into L-AHG and agarotriose, and the intracellular ABG CL4994 depolymerized agarotriose into D-galactose and neoagarobiose. CL5012 further depolymerized neoagarobiose into D-galactose and L-AHG. Also, agarotetraose (the product of α-agarase hydrolysis of agarose by other bacteria) might also be transported into cells via putative transporters. CL4994 depolymerized agarotetraose into D-galactose and neoagarotriose, and CL5012 depolymerized neoagarotriose into L-AHG and agarobiose.

D-galactose as a common monosaccharide can be utilized by microorganisms though the Leloir and DD pathways (Jiang et al., 2020a). The KEGG pathway analysis showed that strain LGH harbored the Leloir and DD pathways, suggesting its metabolic capability of D-galactose (Supplementary Figure 7). Moreover, metabolism of another monosaccharide L-AHG has been reported to be associated with both proteins from *Vibrio* sp. EJY3 (Yun et al., 2015), the 3,6-anhydro-L-galactose dehydrogenase VejAHGD (accession NO. H2IFE7) and the 3,6-anhydrogalactonate cycloisomerase VejACI (accession NO. H2IFX0). In strain LGH, CL4996 shared 44.5% sequence identity to VejAHGD and was present the same aldehyde dehydrogenase family domain (accession NO. pfam00171) as VejAHGD (Supplementary Figure 3). CL5009 shared 55.9% sequence identity to VejACI and harbored the same mandelate racemase like subfamily domain (accession NO. CD03316) as VejACI (Supplementary Figure 3). Both transcription levels of CL4996 and CL5009 increased after strain LGH utilized NAOSs for growth (Figure 1D). Thus, we attempted to assay the functions of CL4996 and CL5009 by gas chromatography mass spectrometry (GC-MS) analysis. The results showed that CL4996, like VejAHGD, had capability to dehydrogenate L-AHG into 3,6-anhydrogalactonate (AHGA) and CL5009 like VejACI was able to isomerize AHGA into 2-keto-3-deoxygalactonate (KDgal) (Supplementary Figure 8). It has been reported that KDgal could be further metabolized through DD pathway in some bacteria (Wong and Yao, 1994; Yun et al., 2015).

**FIGURE 6:**
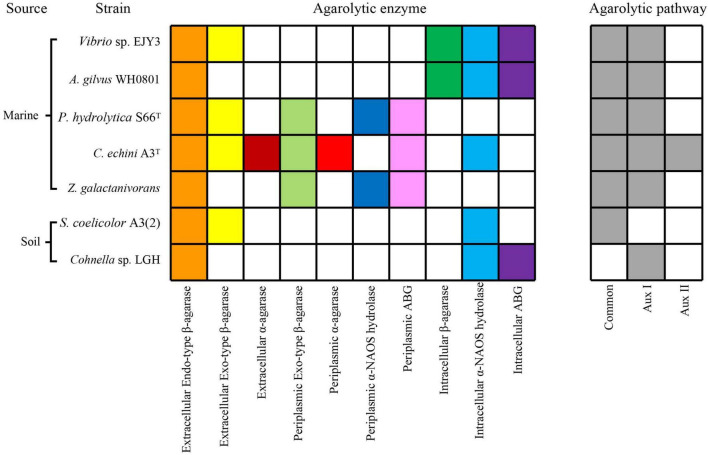
Comparative analysis of characterized agarolytic pathway in bacteria. Colored boxes indicated that bacteria harbored agarolytic enzyme or agarolytic pathway, white boxes indicated that agarolytic enzyme or agarolytic pathway were absent in bacteria.

We summarized and compared seven characterized agarolytic pathways in different bacteria (Figure 6). We noticed that only strain *C*. *echini* A3^T^ harbored Aux II pathway, since it possessed α-agarase which had been demonstrated rare in a natural environment (Pathiraja et al., 2021). Interestingly, we also noticed that a common agarolytic pathway was present in five agar-degrading bacteria along with various auxiliary agarolytic pathways to depolymerize agarose (Schultz-Johansen et al., 2018; Yu et al., 2020; Pathiraja et al., 2021). While strain *Streptomyces coelicolor* A3(2) and strain LGH only harbored one agarolytic pathway. *S. coelicolor* A3(2) harbored the common agarolytic pathway but lacked the auxiliary agarolytic pathways (Jiang et al., 2020b). Strain LGH could not depolymerize neoagarotetraose through a common pathway which other six reported agarolytic bacteria all harbored, since two endo-type β-agarases AgaW and CL5055 could not hydrolyze neoagarotetraose into neoagarobiose. Thus, strain LGH not only harbored novel agarolytic enzymes but also depolymerized agarose in an auxiliary pathway. These results show that the various agarolytic pathway in different bacteria suggest the diversity of agarose depolymerization in a natural environment.

It is interesting that the algal polysaccharide agarose cannot be commonly found in terrestrial environments as there have been increasing reports about agar-degrading bacteria isolated from terrestrial environments in recent years (Li et al., 2015; Pluvinage et al., 2018; Thaller et al., 2018). We noticed that the increasing production of cultured algae is a phenomenon that cannot be ignored. The global production of aquatic plants and algae has tripled from 10 Mt of wet biomass in 2,000 to more than 32 Mt in 2017 (Naylor et al., 2021). Meanwhile, the agarose was widely used in various industrial and experimental applications due to its stabilizing properties (Jiang et al., 2020a). Thus, a large amount of stable agarose has probably been imported into terrestrial environments. Terrestrial bacteria that could utilize agarose as a carbon resource might acquire advantages in competition. This might be a potential reason that some terrestrial bacteria harbored the ability to utilize agarose.

## Data Availability Statement

The datasets presented in this study can be found in online repositories. The names of the repository/repositories and accession number(s) can be found in the article/Supplementary Material.

## Author Contributions

GL and JW designed the studies. GL, RG, SW, and JW analyzed the genome sequence. GL, RG, SC, and JL cloned and purified enzymes. GL and SW performed the qPCR analyses. GL, WX, ZZL, XS, JX, QZ, and ZHL characterized the enzymatic properties. GL wrote the manuscript. JW, ZW, and FH revised the manuscript. All authors contributed to the article and approved the submitted version.

## Conflict of Interest

The authors declare that the research was conducted in the absence of any commercial or financial relationships that could be construed as a potential conflict of interest.

## Publisher’s Note

All claims expressed in this article are solely those of the authors and do not necessarily represent those of their affiliated organizations, or those of the publisher, the editors and the reviewers. Any product that may be evaluated in this article, or claim that may be made by its manufacturer, is not guaranteed or endorsed by the publisher.

## Footnotes

1
https://bcb.unl.edu/dbCAN2/blast.php
