# Supplementary material for: Characterization of Agarolytic Pathway in a Terrestrial Bacterium Cohnella sp. LGH
Source: Front Microbiol. 2022 Mar 31;13:828687. doi: 10.3389/fmicb.2022.828687 (PMC9008576; doi:10.3389/fmicb.2022.828687)
Supplement: Supplementary file 1 [file Data_Sheet_1.PDF]

Table S1 Effects of different regents on activity of CL5012.

Reagents	Concentration(mM)	Relative activity(% ± S.D.)
CK		100±1.02
Na^{+}	10	98.83±2.76
K^{+}	10	98.10±1.44
Mg^{2+}	10	82.28±0.58
Mn^{2+}	10	81.70±1.34
Zn^{2+}	10	148.22±1.21
Ca^{2+}	10	68.52±0.84
Cu^{2+}	10	150.12±4.73
Fe^{2+}	10	107.66±1.99
Fe^{3+}	10	108.13±0.56
EDTA	10	*N.D.*
SDS	10	*N.D.*

N.D.: No enzyme activity was detected

Table S2 Effects of different regents on activity of CL4994.

Reagents	Concentration(mM)	Relative activity (% ± S.D.)
CK		100±1.73
Na^{+}	10	100.58±0.87
K^{+}	10	99.86±1.26
Mg^{2+}	10	100.52±2.27
Mn^{2+}	10	93.02±1.42
Zn^{2+}	10	104.00±2.12
Ca^{2+}	10	85.77±0.79
Cu^{2+}	10	*N.D.*
Fe^{2+}	10	88.14±3.21
Fe^{3+}	10	70.74±2.03
EDTA	10	92.80±1.89
SDS	10	*N.D.*

N.D.: No enzyme activity was detected

Table S3 Effects of different regents on activity of CL5055.

Reagents	Concentration(mM)	Relative activity(% ± S.D.)
CK		100%±0.18
Na^{+}	10	97.13%±0.47
K^{+}	10	98.53%±1.15
Mg^{2+}	10	81.83%±2.13
Cu^{2+}	10	*N.D.*
Zn^{2+}	10	89.23%±1.42
Ca^{2+}	10	86.99%±0.37
Mn^{2+}	10	86.13%±0.89
Ba^{2+}	10	55.47%±0.74
Fe^{3+}	10	54.78%±0.99
Urea	10	103.01%±0.22
SDS	10	*N.D.*
EDTA	10	81.09%±0.68
DTT	10	128.34%±1.21

N.D.: No enzyme activity was detected

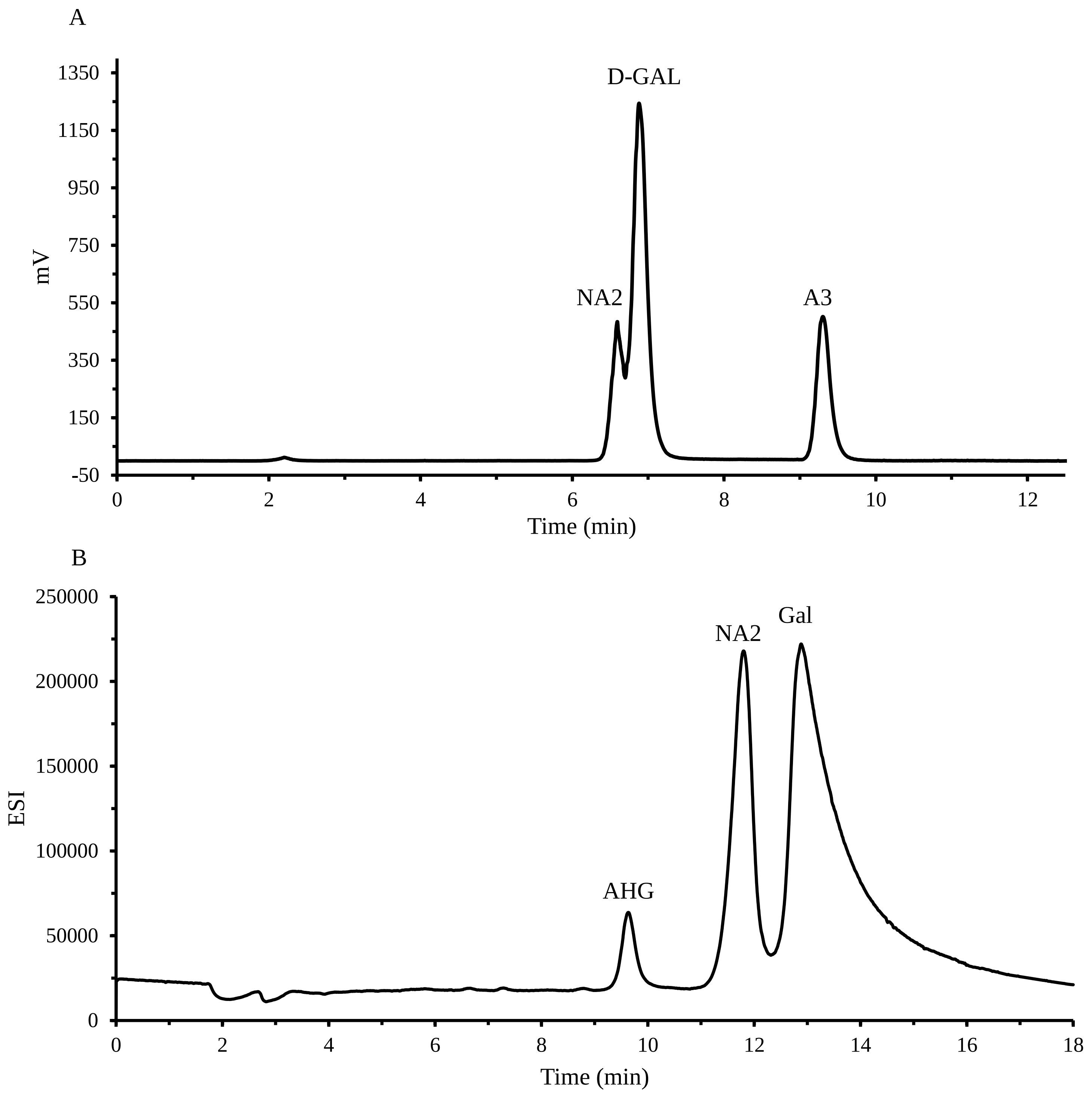

Fig S1 HPLC analysis of hydrolysis products of CL4994 acting on agarotriose (A) and LC-MS analysis of hydrolysis products of CL5012 acting on neoagarobiose (B). AHG, 3,6-anhydro-L-galactose; A3, agarotriose; Gal, D-galactose; NA2, neoagarobiose.

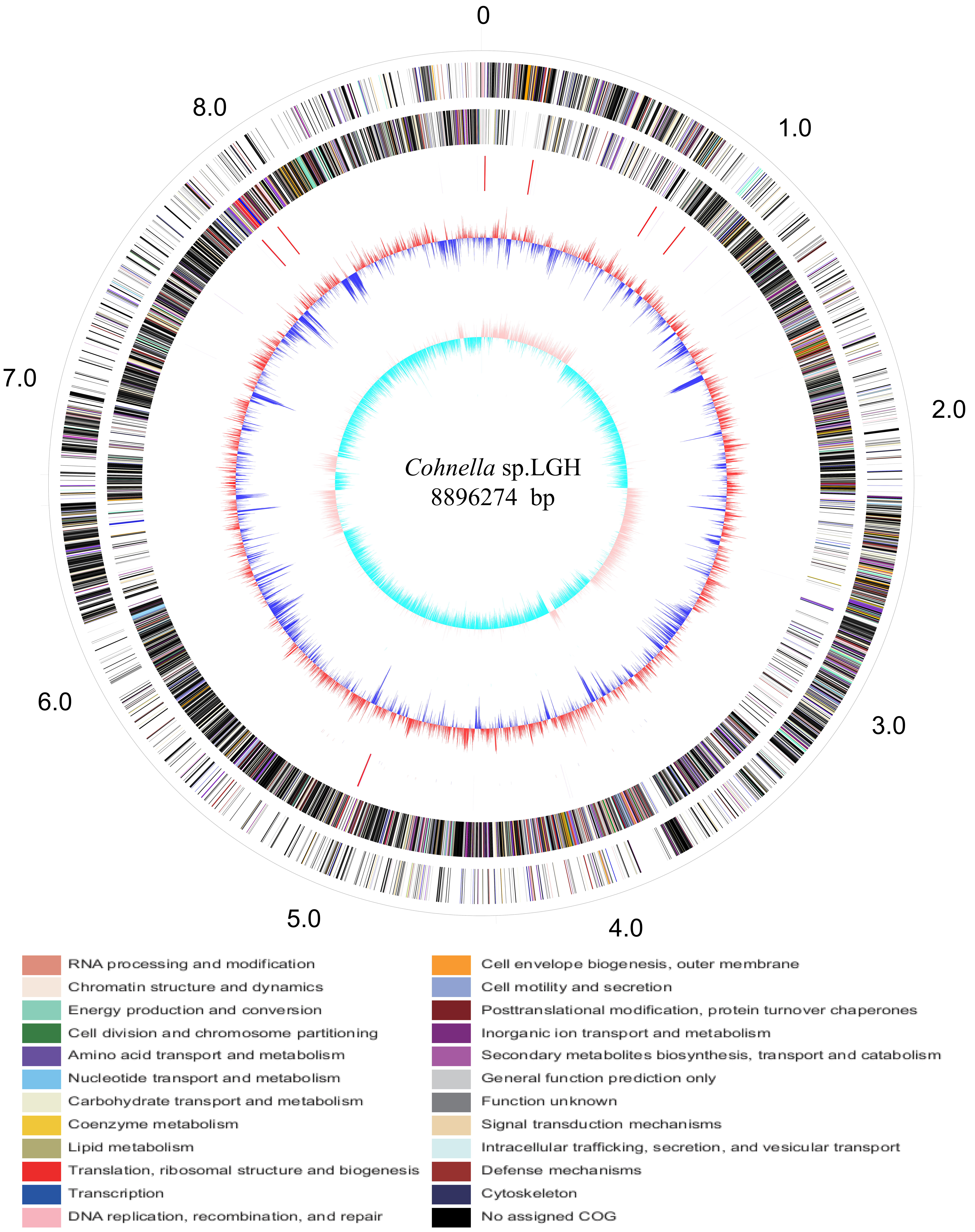

Fig S2 Complete genomic circle map of *Cohnella* sp. LGH.

A

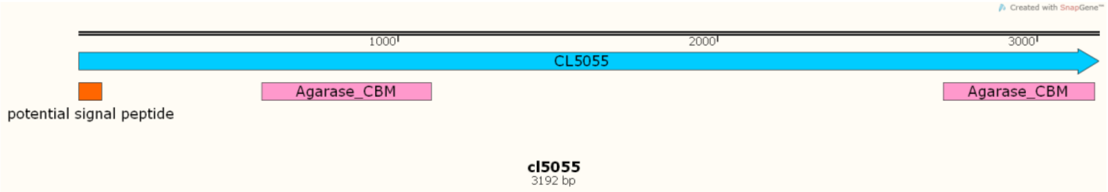
Created with SnapGene
1000
2000
3000
CL5055
Agarase_CBM
Agarase_CBM
potential signal peptide
cl5055
3192 bp

B

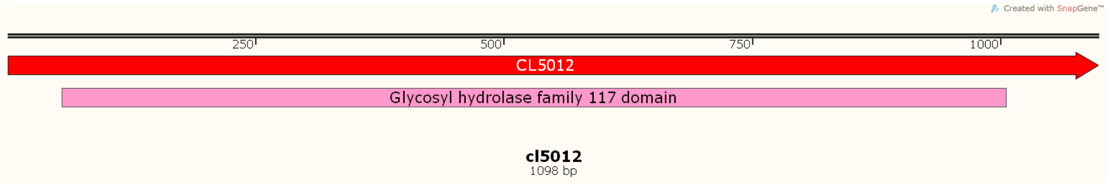
Created with SnapGene
250
500
750
1000
CL5012
Glycosyl hydrolase family 117 domain
cl5012
1098 bp

C

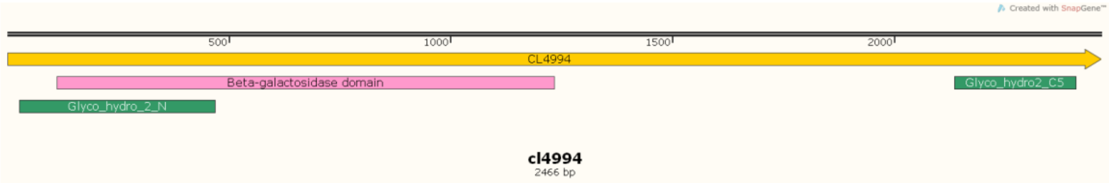
Created with SnapGene
500
1000
1500
2000
CL4994
Beta-galactosidase domain
Glyco_hydro2_C5
Glyco_hydro_2_N
cl4994
2466 bp

D

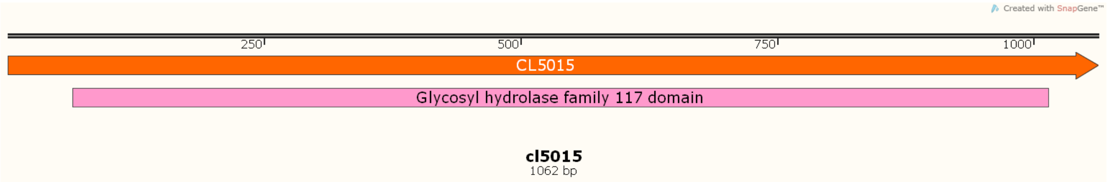
Created with SnapGene
250
500
750
1000
CL5015
Glycosyl hydrolase family 117 domain
cl5015
1062 bp

E

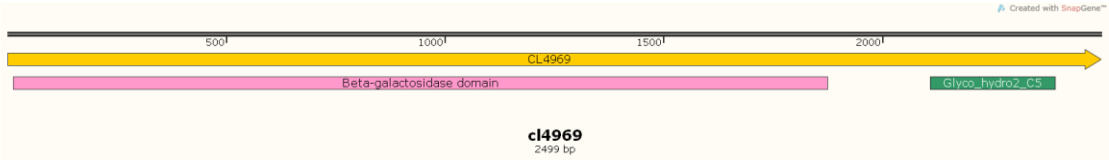
Created with SnapGene
500
1000
1500
2000
CL4969
Beta-galactosidase domain
Glyco_hydro2_C5
cl4969
2499 bp

F

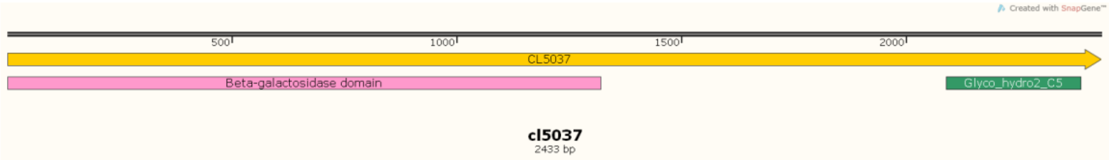
Created with SnapGene
500
1000
1500
2000
CL5037
Beta-galactosidase domain
Glyco_hydro2_C5
cl5037
2433 bp

G

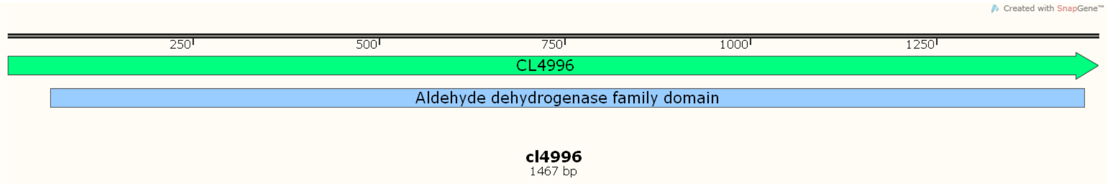
Created with SnapGene
250
500
750
1000
1250
CL4996
Aldehyde dehydrogenase family domain
cl4996
1467 bp

H

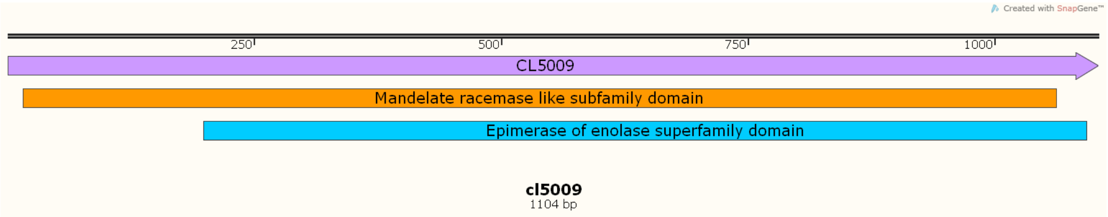

Fig S3 Sequence analysis of CL5055 (A), CL5012 (B), CL5015 (C), CL4994 (D), CL4969 (E), CL5037 (F), CL4996 (G) and CL5009 (H). The signal peptide was predicted by SignalP-5.0 server, (http://www.cbs.dtu.dk/services/SignalP/), conserved domain was by BLASTP (https://blast.ncbi.nlm.nih.gov/Blast.cgi).

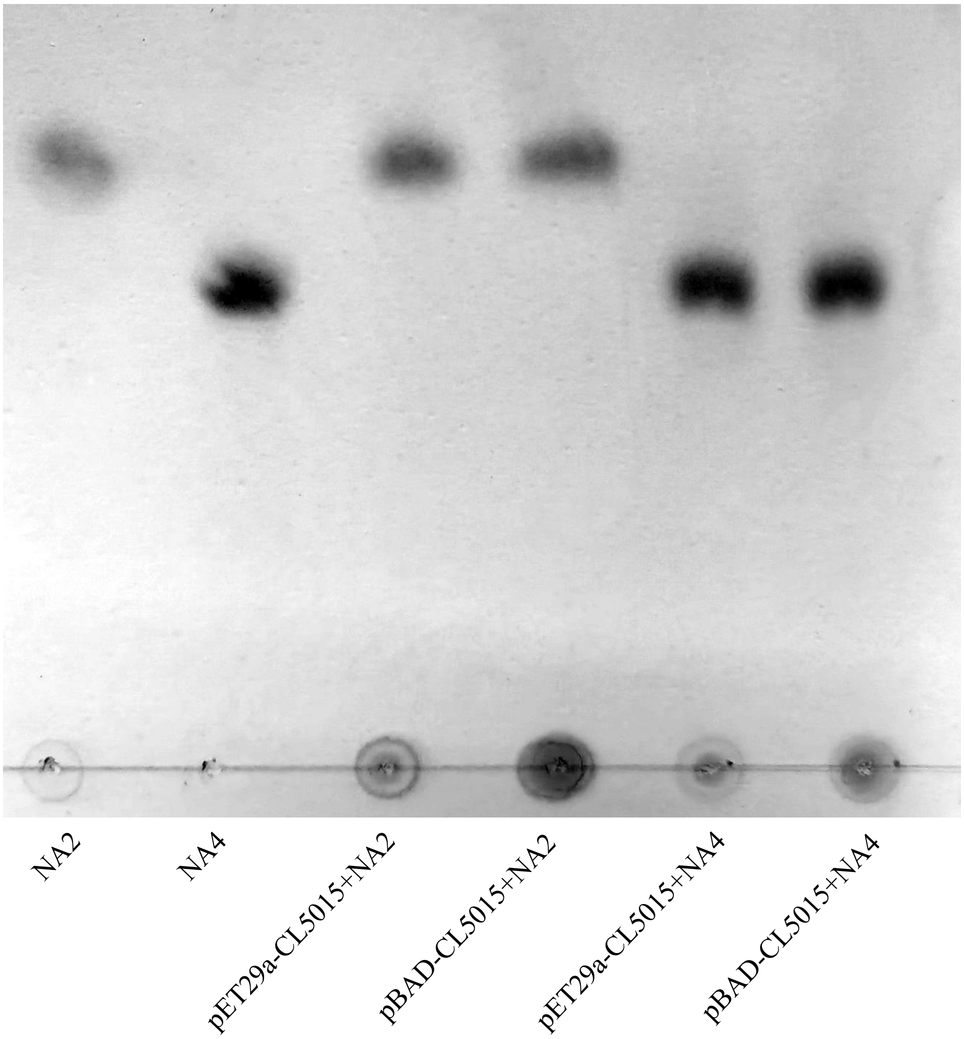

Fig S4 TLC analysis of hydrolytic products of pET29a-CL5015 and pBAD-CL5015 incubated with NAOSs (neoagarobiose and neoagarotetraose), respectively. NA2, neoagarobiose; NA4; neoagarotetraose.

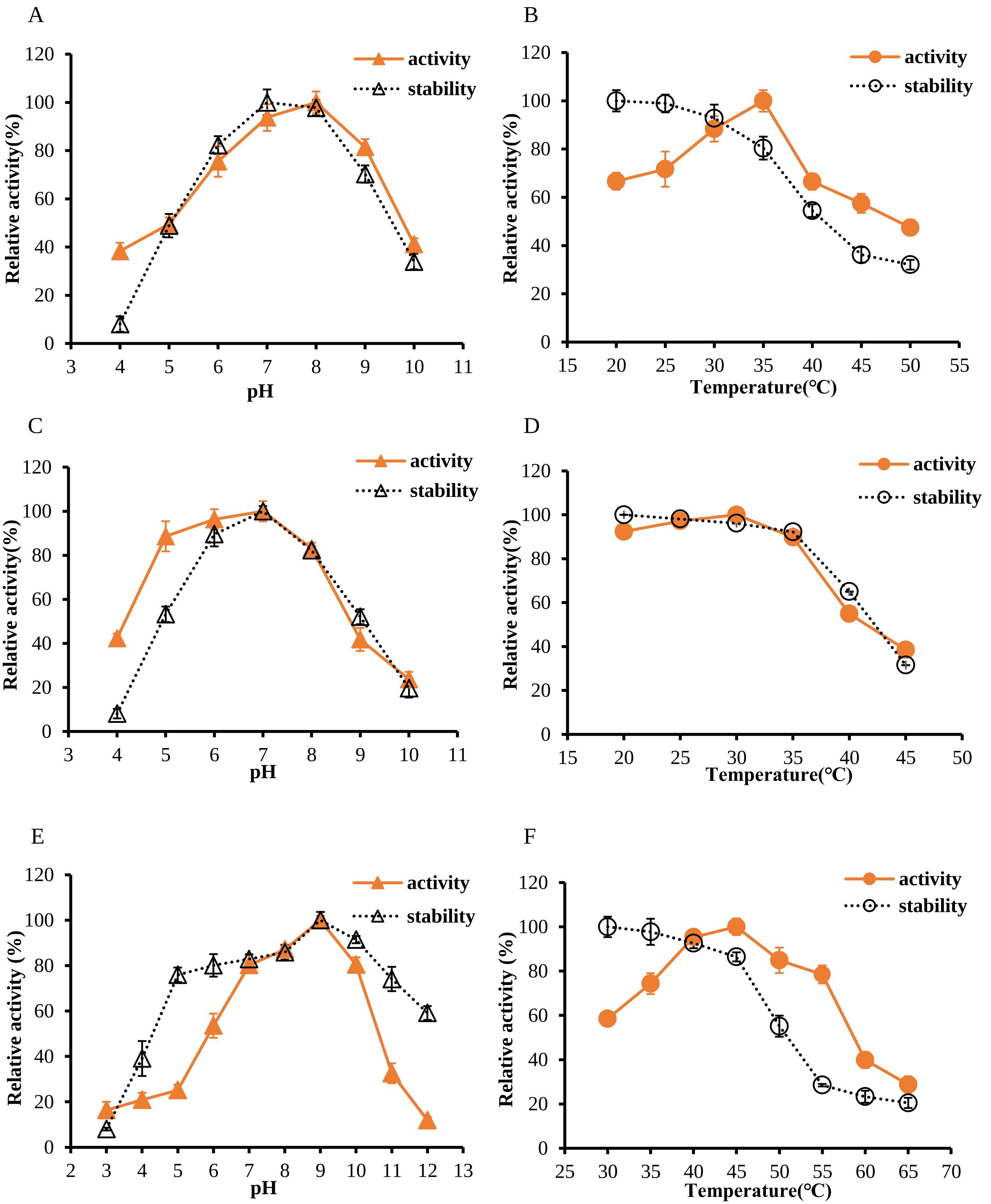

Fig S5 Biochemical properties of CL5055, CL5012 and CL4994. A, Effect of pH on enzymatic activity of the CL5012. B, Effect of temperature on enzymatic activity of CL5012. C, Effect of pH on enzymatic activity of CL4994. D, Effect of temperature on enzymatic activity of CL4994. E, Effect of pH on enzymatic activity of CL5055. F, Effect of temperature on enzymatic activity of CL5055.

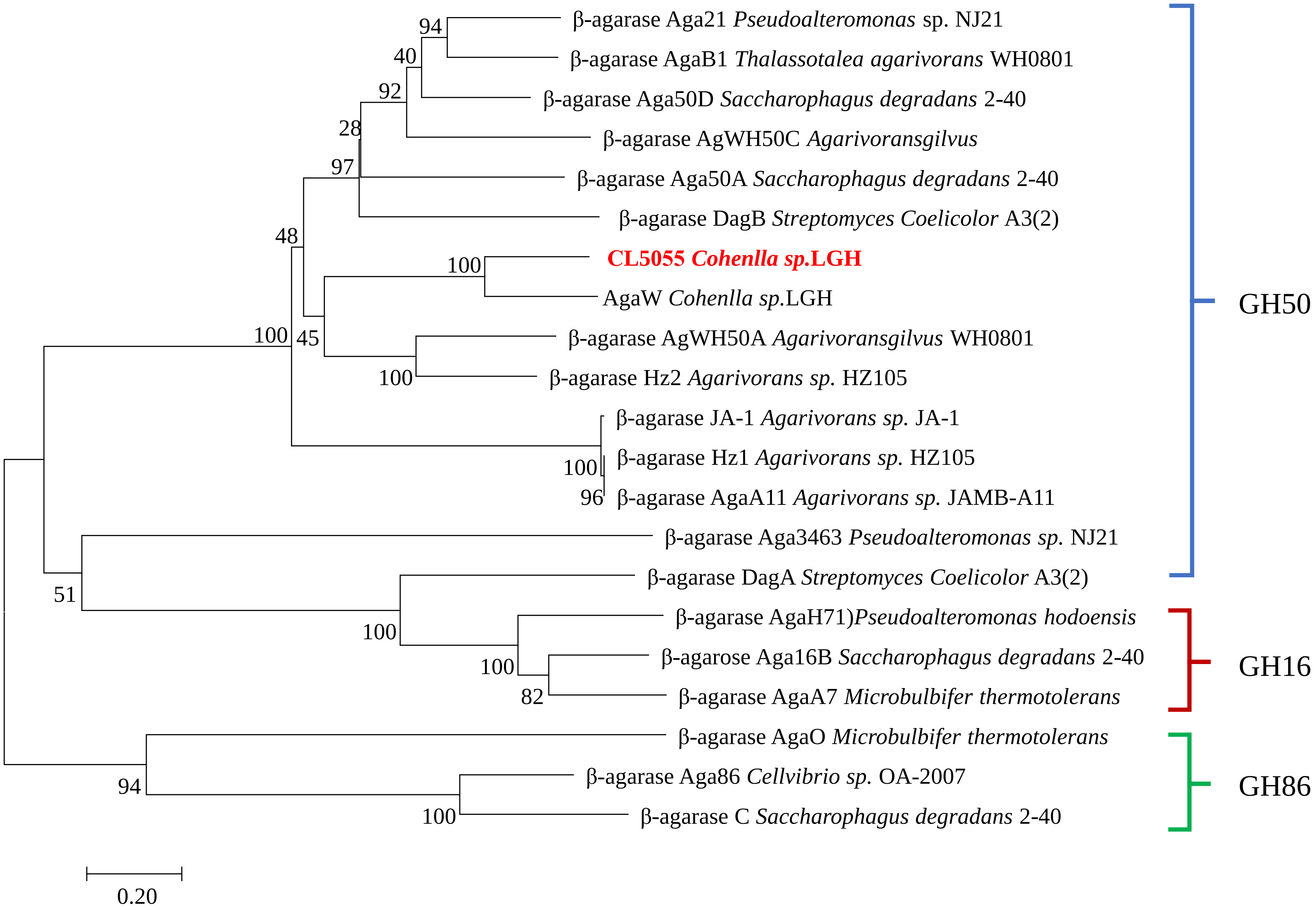

Fig S6 Phylogenetic tree of CL5055. The phylogeny was analyzed by MEGA version 5.2 Software. Distances were calculated using the Kimura two-parameter distance model. Unrooted trees were built by the Neighbor Joining method. The dataset was bootstrapped 1,000 times. The result illustrated that CL5055 exhibited highest sequence similarity to GH50 family.

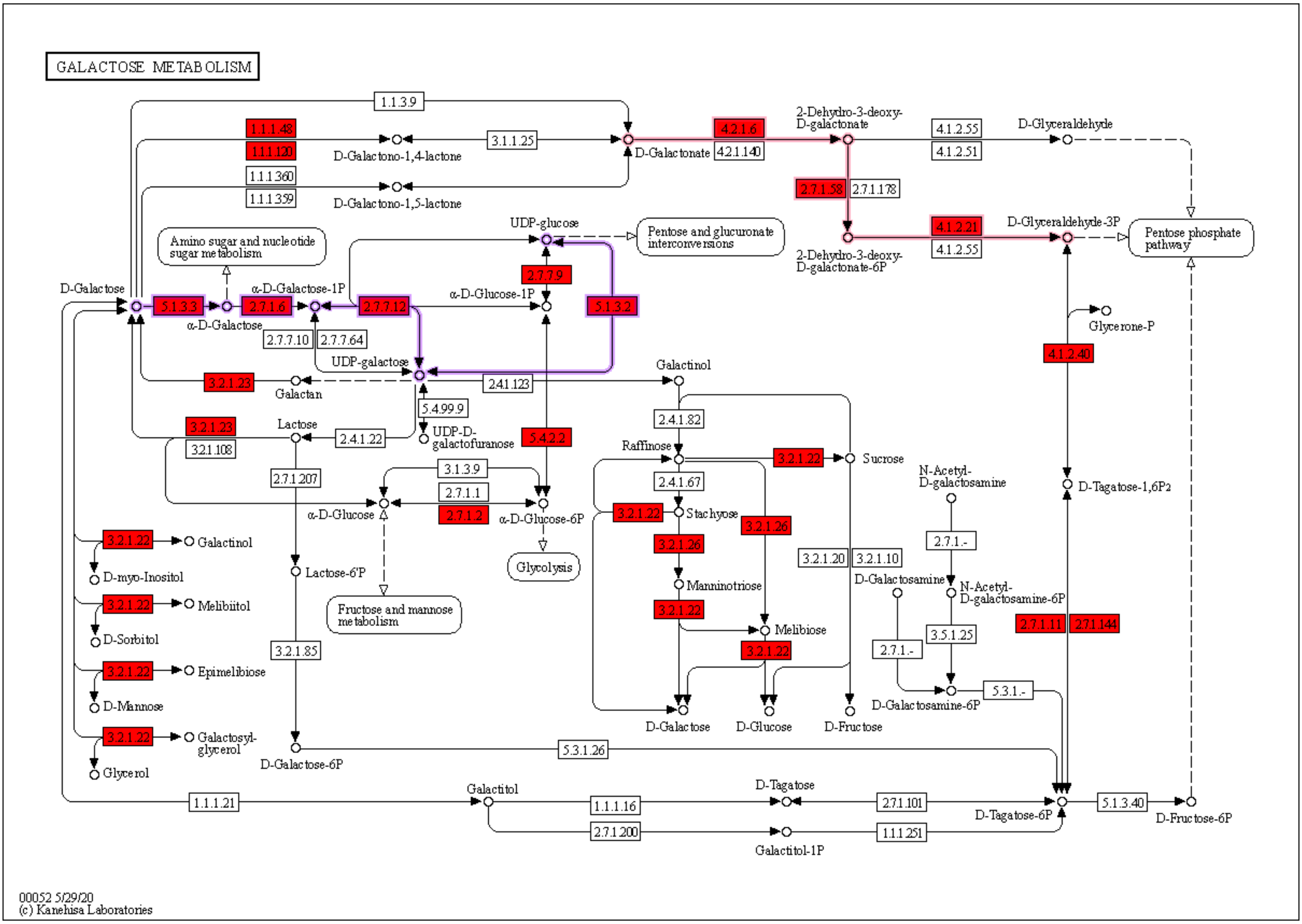

Fig S7 KEGG analysis of galactose metabolism pathway in *Cohnella* sp. LGH. Boxes in red, strain LGH harbored this type enzyme, line in purple, Leloir pathway; line in pink, DeLey-Doudoroff pathway.

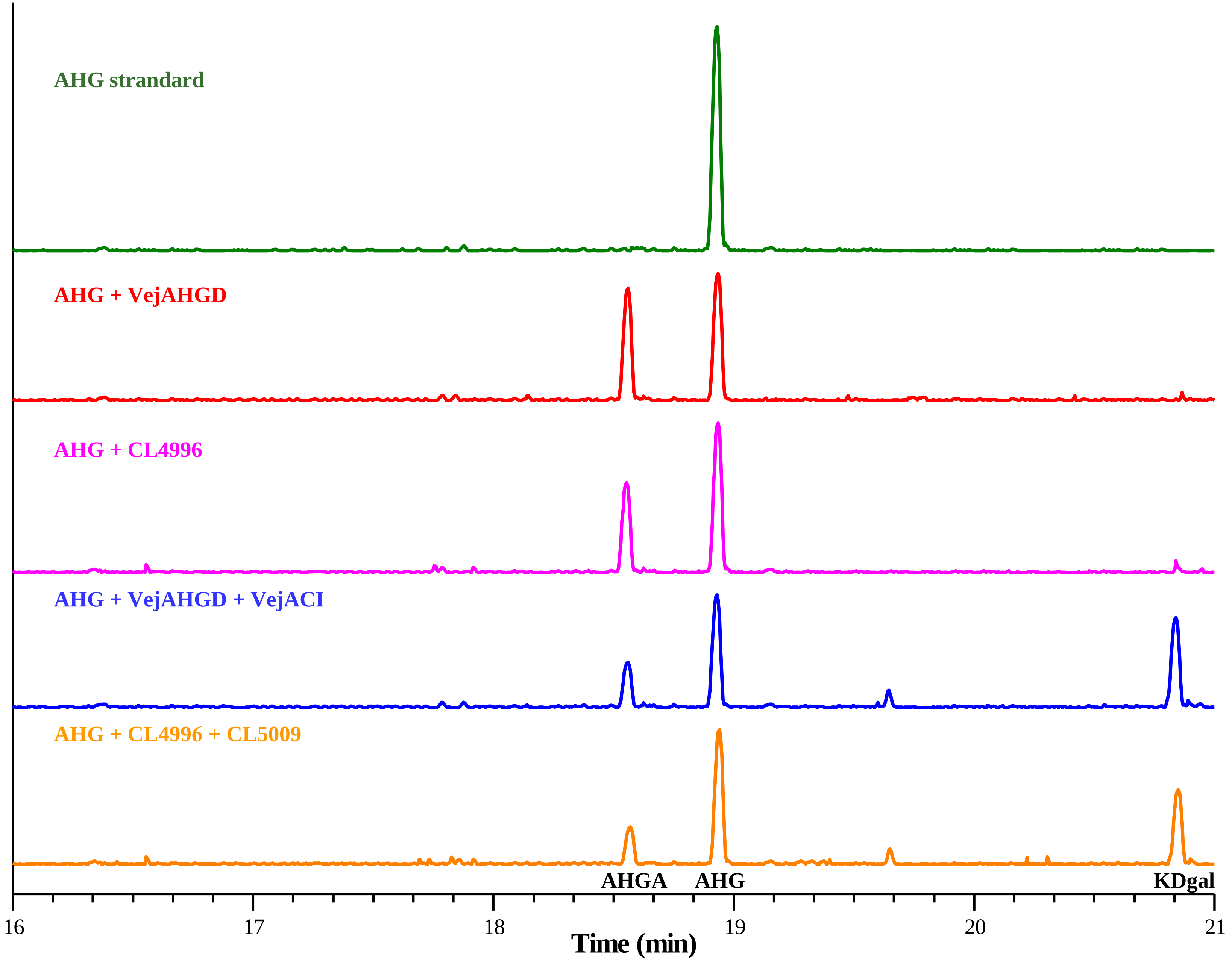

Fig S8 GC-MS analysis of the products of L-AHG by CL4996 and mixture of CL4996 and CL5009. VejAHGD and VejACI were used as positive control.
